# Synchronous twin track percutaneous nephrolithotomy: A preliminary experience

**DOI:** 10.4103/0970-1591.38618

**Published:** 2008

**Authors:** S. V. Kandasami, R. Krishna Rao, M. Arul, M. S. Ranganath, T. Krishna Prasad

**Affiliations:** Department of Urology, Vedanayagam Hospital, Coimbatore, Tamil Nadu, India; 1 Department of Urology, Vikram Hospital and Heart Care, Mysore, Karnataka, India

**Keywords:** Percutaneous nephrolithotomy, staghorn calculi, synchronous

## Abstract

Percutaneous nephrolithotripsy is the treatment of choice for large renal calculi. Total stone clearance as the treatment goal remains elusive, despite staged procedures and multiple tracks. There is also the morbidity of multiple sittings and tracks. We investigated the feasibility of a synchronized approach to clearing these difficult stones with two teams operating through two or more tracks in tandem and also the ergonomic and logistic issues involved.

## MATERIALS AND METHODS

**Figure 1:**
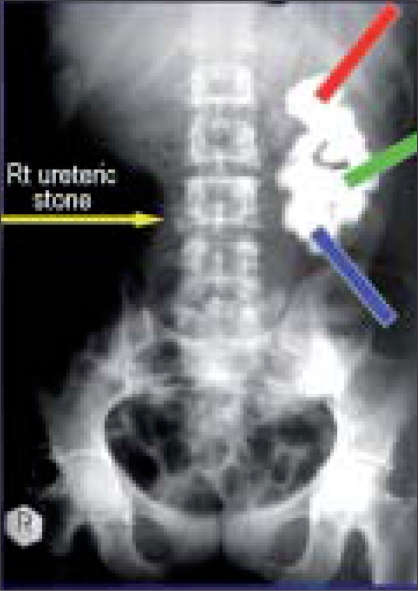
Preoperative KUB illustrating track placement

**Figure 2:**
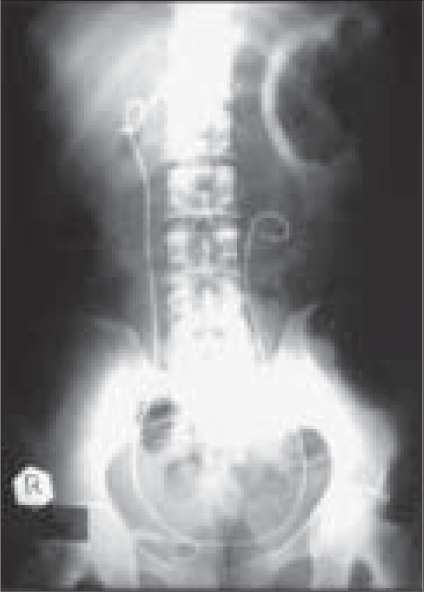
After repeat PCNL

Four men and one woman (average age: 48 years) underwent this procedure between March 2006 and February 2007. Their staghorn stones ranged in size from 5.3 cm × 3.6 cm to 3.2 cm × 2.5 cm. Two surgeons performed the procedure using two sets of Percutaneous nephrolithotomy (PCNL) equipment; three patients required three tracks while two required two tracks [Figures 1 and 2].

## RESULTS

All patients withstood the procedure well, mean operating time ranged from one and a half hours to two and a half hours (average two hours). Three patients required a second look procedure for residual fragments. On completion of the treatment three patients had residual fragments of size less than 8 mm. There were no significant postoperative complications; two patients had fever which settled with conservative treatment. Average hospital stay was five days.

## DISCUSSION

Percutaneous nephrolithotomy is now the standard technique for staghorn renal calculi.[1] Improved instrumentation and technical advancement have enabled urologists to perform percutaneous stone removal with increasing efficacy and decreasing complications.[2] There is, however, a decrease in the overall stone-free rate, an increase in the complication rate, the secondary procedure rate, the mean operative time and the need for multiple tracts, with increasing stone surface area.[3]

Our indications for this approach were large stone surface area, complex pelvicalyceal anatomy where two or more tracks were deemed necessary to clear the calculi.

Synchronous PCNL requires two experienced surgeons working together, assistants and nursing staff familiar with the technique and two complete sets of instruments including telescopes, cameras, monitors, energy sources and grasping instruments. A solitary C-arm is adequate as the imaging modality, but necessitates preliminary planning of track placement which has to be done sequentially.

**Figure 3:**
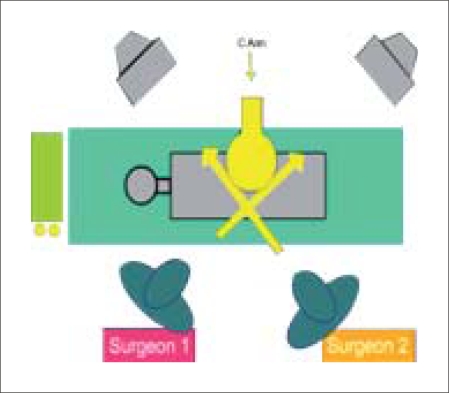
OT setup illustration

Tracks are placed ideally in the superior and inferior calyx at an obtuse angle in relation to each other such that the telescopes with all the attached cables and irrigation sets do not get entangled. The single C-arm limits the angles from which each surgeon can view the position of guide wires and scopes, so meticulous planning and cooperation are paramount [Figure 3].

The advantages when compared to the single-team PCNL are manifold. Operating time and blood loss are reduced; there are multiple views of the stone/s.

To reduce intrarenal pressure and irrigating fluid absorption we use the largest available (32 Fr) Amplatz thus ensuring free flow of effluent between the sheath and the nephroscope (27 Fr). The theoretical risk of extensive renal laceration due to counter traction and aggressive manipulation is obviated by close coordination between the two operating teams. Postoperative Intravenous Urogram in four patients revealed no anatomical evidence of renal damage.

This first description of Synchronous Twin Track PCNL in a single renal unit by two operating teams indicates that the procedure is feasible, safe and worthy of further evaluation.

## CONCLUSION

Synchronous Twin track PCNL is a potentially ideal operation for complete clearance of large, complex staghorn calculi. Future areas for investigation include quantifying blood loss, intrarenal pressures, irrigant absorption and renal damage. The need for two experienced teams operating in tandem with two complete sets of instruments would limit the technique to hospitals where these are available.
